# Clozapine reduces infiltration into the CNS by targeting migration in experimental autoimmune encephalomyelitis

**DOI:** 10.1186/s12974-020-01733-4

**Published:** 2020-02-12

**Authors:** Katharina Robichon, Vimal Patel, Bronwen Connor, Anne Camille La Flamme

**Affiliations:** 1 School of Biological Sciences, Victoria University of Wellington, Wellington, New Zealand; 2 Centre for Biodiscovery Wellington Victoria University of Wellington, Wellington, New Zealand; 3 Department of Pharmacology and Clinical Pharmacology, Centre for Brain Research, School of Medical Science, Faculty of Medical and Health Science, University of Auckland, Auckland, New Zealand; 4 Malaghan Institute of Medical Research, Wellington, New Zealand

**Keywords:** EAE, MS, Clozapine, Neuroinflammation, Migration

## Abstract

**Background:**

Atypical antipsychotic agents, such as clozapine, are used to treat schizophrenia and other psychiatric disorders by a mechanism that is believed to involve modulating the immune system. Multiple sclerosis is an immune-mediated neurological disease, and recently, clozapine was shown to reduce disease severity in an animal model of MS, experimental autoimmune encephalomyelitis (EAE). However, the mode of action by which clozapine reduces disease in this model is poorly understood.

**Methods:**

Because the mode of action by which clozapine reduces neuroinflammation is poorly understood, we used the EAE model to elucidate the in vivo and in vitro effects of clozapine.

**Results:**

In this study, we report that clozapine treatment reduced the infiltration of peripheral immune cells into the central nervous system (CNS) and that this correlated with reduced expression of the chemokines CCL2 and CCL5 transcripts in the brain and spinal cord. We assessed to what extent immune cell populations were affected by clozapine treatment and we found that clozapine targets the expression of chemokines by macrophages and primary microglia. Furthermore, in addition to decreasing CNS infiltration by reducing chemokine expression, we found that clozapine directly inhibits chemokine-induced migration of immune cells. This direct target on the immune cells was not mediated by a change in receptor expression on the immune cell surface but by decreasing downstream signaling via these receptors leading to a reduced migration.

**Conclusions:**

Taken together, our study indicates that clozapine protects against EAE by two different mechanisms; first, by reducing the chemoattractant proteins in the CNS; and second, by direct targeting the migration potential of peripheral immune cells.

## Background

Multiple sclerosis (MS) is an autoimmune disease characterized by continuous infiltration of autoreactive T cells and other inflammatory immune cells from the periphery into the central nervous systems (CNS). The clinical symptoms of MS are attributed to the inflammatory lesions in the CNS white matter regions leading to sensory dysfunction and loss of motor control. During MS, the pathological recruitment of myelin-specific CD4 T cells and other immune cells into the CNS results in demyelination of the neuronal axons [1, 2]. This immune cell infiltration and demyelination can be studied in the well-established experimental autoimmune encephalomyelitis (EAE) MS animal model [3]. Current therapeutic strategies including glatiramer acetate, interferon-β, or natalizumab show only limited effects on the different forms of MS [4–7]. Most of these medicines are immunomodulatory agents acting in the circulating compartment due to the low capacity to cross the intact blood-brain barrier [8, 9].

Under physiological conditions, the blood-brain barrier protects the CNS against leukocyte infiltration. Understanding which factors regulate the initial and early infiltration into the CNS during MS is central to the development of treatment strategies; however, the specific factors that regulate leukocyte trafficking and accumulation in the CNS are not fully defined [10]. Migration of activated leukocytes and macrophages is controlled by several different proinflammatory chemotactic cytokines, called chemokines [11]. These are produced and released locally and diffuse into the bloodstream thereby attracting leukocytes to the site of inflammation. Monocyte chemoattractant protein 1 (MCP-1) or CCL2 and regulated upon activation normal T cell expressed and secreted (RANTES) or CCL5 belong to the family of C-C chemokines involved in the recruitment of monocytes, macrophages, and activated lymphocytes to the site of expression [12] and mediate leukocyte adhesion to epithelial cells [13]. CCL2 and CCL5 are expressed by a wide array of different cell types constitutively within the CNS or secreted by infiltrating blood-derived macrophages upon their migration into the CNS. Interestingly, the production of CCL2, CCL3, and CCL5 in the CNS has been associated with acute disease symptoms in rat and mice [14, 15]. Given the evidence that CCL2 and CCL5 are highly involved in the regulation of EAE, modulating the expression or function of CCL2 and CCL5 attracts much attention as a potential therapy for MS [16, 17]. Drugs that would target CCL2 and CCL5 expression directly would be highly beneficial.

Clozapine is a small molecule drug that readily crosses the blood-brain barrier [18], and is an atypical antipsychotic agent used in the treatment of neuropsychiatric disorders like schizophrenia [19]. Neuropsychiatric disorders are increasingly recognized as being associated with inflammation with elevated expression of inflammation markers in the CNS. The atypical antipsychotic drug clozapine has been shown to be effective at reducing disease in EAE in a prophylactic [20] and therapeutic manner [21]. The ability of clozapine to reduce disease severity, however, was found not to be mediated by a direct effect on myelin-specific CD4 T cells [22]. Therefore, a different mechanism of action is believed to underlie the beneficial effects of clozapine treatment in EAE.

In this study, we investigated the underlying mechanism by which clozapine reduces disease onset and severity by evaluating the initial infiltration of immune cells into the CNS to determine if immune cell migration is the functional target of clozapine.

## Methods

### Animals

Female C57BL/6 J mice were bred and housed in the animal facility at Victoria University of Wellington, New Zealand and used between 8 and 12 weeks of age.

### Ethics statement

All of the experiments with animals were carried out in the School of Biological Sciences Animal Facility at Victoria University of Wellington and were approved by the Victoria University of Wellington Animal Ethics Committee (2014-R23).

### EAE induction and treatments

Mice were immunized s.c. in the rear flanks with myelin oligodendrocyte glycoprotein (MOG)_35–55_ peptide (50 μg/mouse; Genescript, Piscataway, NJ) in complete Freund’s adjuvant (Sigma, St. Louis, MO) containing 500 μg/mouse *Mycobacterium tuberculosis* (Fort Richard, Auckland, New Zealand). In addition, mice were injected i.p. with pertussis toxin (200 ng/mouse; List Biochemicals, Campbell, CA) on days 0 and 2. Mice were weighed and scored daily as follows: 0, normal; 1, partial tail paralysis; 2, full tail paralysis; 3, paralysis in one hind limb; 4, paralysis in both hind limbs; and 5, moribund. One day before immunization, mice drinking water was changed to 60 mg/kg/day Clozapine (kindly supplied by Douglas Pharmaceuticals Ltd. (Auckland, New Zealand)) or vehicle (0.1 M acetic acid) in the drinking water. Mice were treated with clozapine or vehicle over the whole time of the experiment. Following CO_2_ euthanasia, brains, spinal cord, spleens, and blood were isolated and processed into a single-cell suspension.

### Primary cell isolation into single-cell suspension

Spinal cord was minced and incubated with collagenase type II for 30 min at 37 °C. Remaining clumps were broken up using at pipette and cell suspension was passed through a 70 μm cell strainer and centrifuged at 760 g for 5 min. Brain was mashed through a 70 μm cell strainer and centrifuged at 760 g for 5 min. Spinal cord and brain cell pellet were resuspended in 37% Percoll™ gradient and centrifuged 30 min at 760 g without brakes. Myelin layer was removed, supernatant discharged, and pellet resuspended for cell counting. Lymph nodes were mashed through a 70-μm cell strainer and centrifuged at 760 g for 5 min and cell pellets were resuspended for cell counting.

Spleen was mashed through a 70-μm cell strainer and centrifuged at 760 g for 5 min, pellet was loosen and resuspended in Red Cell Lysis buffer for 2 min. Whole blood was also incubated with Red Cell Lysis buffer for 2 min, wash buffer was added, and samples were centrifuged at 760 g for 5 min. For the blood, lysis step was repeated at least once. Afterwards, cell pellets were resuspended for cell counting.

### Flow cytometry

For the detection of immune cells, the following antibodies were used: CD4-BV421 (RM4-5; Biolegend, San Diego, CA, USA), CD45-BV510 (30-F11; Biolegend), CD25-AF488 (PC61; Biolegend), CD8-PerCPCy5.5 (53-6.7; Biolegend), CD11b-PE-Cy7 (M1/70; Biolegend), CD3-APC-Cy7 (17A2; Biolegend), Ly6C-PE (HK1.4; Biolegend), Ly6G-APC (1A8; Biolegend), CD45R-AF488 (RA3-6B2; BD Bioscience), CCR2-PE (475,301; R&D), CCR5-APC (HM-CCR5; Biolegend), and Gr1-APC-Cy7 (RB6-8C5, Biolegend). Cells were incubated with Fc Block (2.4G2; BD Biosciences) for 15 min prior to staining with fluorescently labeled antibodies for 30 min on ice. Flow cytometry was performed on a BD FACS Canto II (BD Biosciences) and analyzed using Flowjo software version 10.1 (Treestar Inc., Ashland, OR, USA).

### In-vivo migration assay

C57BL/6 mice were treated with 60 mg/kg/day Clozapine (kindly supplied by Douglas Pharmaceuticals Ltd. (Auckland, New Zealand)) or vehicle (0.1 M acetic acid) in the drinking water for 7 days. On the following day, mice were injected s.c. with either 10 μg/ml of the chemokine CCL5 (RANTES; Peprotech) or 1 μg/ml of the chemokine CCL2 (MCP-1, Peprotech) in 50 μl dPBS (Invitrogen, USA) into the left hind flanks of the mice, while an equal volume of dPBS (vehicle) was injected into the right hind flank. Eighteen hours following hind flank injections, cells from the draining lymph nodes were isolated, counted, and processed for flow cytometry analysis, as described above.

### Isolation and in-vitro culture of cells

Primary microglia (pooled from four to five mice per experiment) were derived as described previously [23], purity was checked by flow cytometry staining for CD45^low^CD11b^+^CD3^−^ and was on average 76%. Primary microglia were seeded at 5 × 10^4^ cells/well in microglia media and 10 ng/ml macrophage colony-stimulating factor (M-CSF) (ProSpec) in a flat-bottomed 96-well plate and cultured for 4 weeks with media changes every 3–4 days. After 4 weeks in culture, the mature, adherent microglia in the 96-well plates were stimulated with or without LPS (200 ng/ml, Sigma) and pre-treated or co-treated with 20 μM clozapine.

Bone marrow-derived macrophages (BMMO) were isolated and cultured as described [24]. BMMO were either derived with 5 ng/ml granulocyte-macrophage colony-stimulating factor (GM-CSF) (Peprotech) and 5 ng/ml IL3 (Peprotech) or 10 ng/ml M-CSF for 9 days (ProSpec). BMMO (10^5^/well) were cultured in complete culture media in 96-well plates. GM-CSF-derived BMMO were primed overnight with interferon gamma (IFNγ) (20 U/ml; Peprotech) before stimulating with or without LPS (200 ng/ml, Sigma) and 20 μM or 40 μM clozapine. M-CSF-derived BMMO were primed overnight with IL4 (20 ng/ml; ProSpec) before stimulating with or without LPS (200 ng/ml, Sigma) and 20 μM or 40 μM clozapine.

Primary astrocytes were kindly generated and provided by Matt Rowe (Victoria University of Wellington) using the Neural Tissue Dissociation Kit (T) (Miltenyi Biotech) as per supplied instructions. Microglia, oligodendrocytes, and neurons were sitting on top of the bottom layer of astrocytes. Cells were cultured in specific astrocyte medium. Microglia-containing supernatant and oligodendrocytes/neurons-containing supernatant was mechanically shaken off and discarded. Adhered cells were approximately 90% astrocytes. Cells were stimulated with IFNγ (20 U/ml; Peprotech) overnight before stimulating with or without LPS (200 ng/ml, Sigma) and 20 μM or 40 μM clozapine.

After the treatment, supernatant was frozen at − 20 °C and cells were collected and re-suspended in TRIZOL for RNA analysis and frozen at − 80 °C.

### In-vitro migration assay

To assess cell motility, 120,000 GM-CSF/IL3-derived BMMO were seeded in complete culture medium into wells of the 96-well ORIS plate (Platypus Technologies, WI, USA) containing a round silicon insert set in each well. Cells were incubated overnight to settle before plugs were removed with the supplied tool. Media was carefully aspirated and replaced with fresh media. Each well was inspected using the microscope, noting wells with disturbed exclusion zones: these wells were not used. Images of wells were captured at this time to measure original wound size and used as timepoint 0. Cells were either left unstimulated, stimulated with 25 μM clozapine, or 0.001 μM Latrunculin A (Sigma-Aldrich) as a positive control or the respective vehicles. Plates were incubated for 3 days before images of each well were taken to measure final wound size. Cell viability was assessed via MTT assay at the end of the experiment.

ImageJ running the MRI Wound Healing Tool macro (Montpellier RIO Imaging, Montpellier, France) was used to measure wound size from images using the following script parameters: method: variance; variance filter radius: 5; threshold: 50; radius open: 1; min. size: 10,000. Compound-induced changes to wound closure were assessed using the following equations:

$$ \mathrm{Closure}\ \left(\%\right)=\left(\frac{{\mathrm{wound}\ \mathrm{area}}_{\mathrm{day}0}-{\mathrm{wound}\ \mathrm{area}}_{\mathrm{day}3}}{{\mathrm{wound}\ \mathrm{area}}_{\mathrm{day}0}}\ \right)\times 100 $$

$$ \mathrm{Closure},\mathrm{as}\%\mathrm{vehicle}=\left(\frac{\mathrm{closure}\ {\left(\%\right)}_{\mathrm{test}\ \mathrm{compund}}}{\mathrm{closure}\ {\left(\%\right)}_{\mathrm{vehicle}}}\ \right)\times 100 $$

### RNA analysis

Total mRNA was isolated by re-suspending the cell pellets in TRIZOL reagent and mRNA extraction was performed by using the Direct-zol™ RNA MiniPrep (Zymo Research) according to manufacturer’s protocol. In summary, TRIZOL cell suspension was mixed in 1:1 ratio with 100% absolute ethanol and added on Zymo-Spin™ IIC columns where mRNA bounds to membrane. Column was washed and DNA was denatured by DNase I digestion on the column. After washing with two buffers with decreasing salt concentration, mRNA was eluted in 30 μl RNase and DNase free water and stored at − 80 °C for further analysis.

Concentration of total mRNA was determined by measuring absorbance at 260 nm using the Nanodrop 2000 (Thermo Fischer). For cDNA generation, 100 ng of total mRNA was used and transcribed with High-Capacity cDNA Reverse Transcription Kit (Applied Biosystems) according to manufacturer’s instructions. Quantitative real-time PCR (qRT-PCR) was performed using the Platinum® SYBR® Green qPCR SuperMix-UDG (Invitrogen) in combination with the CFX Connect™ Real-Time PCR Detection System (BioRad). Relative quantification was performed by normalizing to the reference gene Cyclophilin A and to healthy vehicle control by using the 2^–ΔΔCT^ (Livak) Method.

### Analysis of cytokines by Milliplex

Mouse brains were isolated, weight, and mashed in RIPA Buffer (500 mg/ml) using a tissue homogenizer. Samples were incubated shaking 30 min at 4 °C, centrifuged 10 min at 10,000 g at 4 °C, and supernatant was analyzed for cytokine expression using the 32-plex MILLIPLEX MAP Mouse Cytokine/Chemokine Magnetic Bead Panel (MCYTOMAG-70K Millipore, Germany) according to manufacturer’s protocol. Briefly, single magnetic beads coupled with specific cytokines were mixed. Then, 50 μl of mixture was added to a 96-well plate and washed twice with Washing Buffer by using the BioPlex Pro™ II Wash Station (BioRad) with the magnetic plate carrier. Further, 50 μl of cytokine standards and samples were loaded on the plate and were incubated for 30 min at RT vertical shaking at 300 rpm by using IKA MTS 2/4 digital shaker (IKA WERKE). Samples and standards were removed by the Wash Station and wells were washed three times with washing buffer. Single specific detection antibodies for each cytokine were mixed and 25 μl of mixture was added to all wells followed by incubation for 30 min at RT vertical shaking at 300 rpm. Before adding 50 μl secondary streptavidin-PE conjugated antibody, wells were washed three times. Incubation with streptavidin-PE was performed for 10 min at RT vertical shaking at 300 rpm followed by three times washing with washing buffer. Further, 125 μl of Assay Buffer was added to each well and plate was shaked vertical for 30 s at 600 rpm. Data acquisition was performed by using the BioPlex 200 System and data analysis by using the BioPlex Manager™ software.

### Monocyte isolation and enrichment

Single cell suspensions from spleens were generated as described above. Monocyte isolation was performed using the EasySep™ Mouse Monocyte Isolation Kit (StemCell Technology) according to manufacturer’s instructions. Briefly, samples were incubated with rat serum before the selection cocktail and the RapidSpheres™ were added. After the incubation time, samples were placed into the magnet and the enriched cell suspension was poured out in a continuous motion. The enriched cell suspension was again placed into the magnet and the higher enriched cell suspension was collected in the same way. Isolated cell purity was check by flow cytometry staining for CD45, CD3, and CD11b. Cells were counted as monocytes that were CD45 and CD11b positive and CD3 negative, purity was 70–90%. Cells were plated at 1 × 10^5^ cells/well in a 96-well plate and used for cAMP measurement.

### Measuring intracellular cyclic AMP

Primary splenocytes were isolated into a single cell suspension and seeded onto a 96-well plate at 1 × 10^6^ cells/well. Splenocytes and isolated monocytes were incubated with stimulation buffer containing IBMX for 30 min prior of stimulation. Cells were either pre-treated with 20 μM clozapine for 1 h following forskolin (6.25 μM, Sigma), CCL2, or CCL5 (100 ng/ml; Peprotech) treatment or co-stimulated with the other treatments for 18 h. Monocyte in addition were co-stimulated with LPS (200 ng/ml, Sigma) for the whole time. Cells were washed with phosphate-buffered saline (PBS) and dried in pure ethanol. Afterwards, lysis buffer was added for 1 h shaking. Cell lysates were stored at − 20 °C until further use. Intracellular cyclic AMP levels were measured using the AlphaScreen Kit (Perkin Elmer) according to manufacturer’s instructions. Briefly, in an OptiPlate (Perkin Elmer), 5 μl acceptor beads were added per well and incubated with 5 μl standards and samples for 30 min prior to adding 15 μl donor beads for 8–12 h in reduced light conditions at room temperature. Plate was measured on an EnSpire multilable plate reader (Perkin Elmer) using standard AlphaScreen settings.

### Statistical analyses

All graphs and statistical analyses were generated using GraphPad Prism 7 (GraphPad Software Inc., La Jolla, CA USA). Comparisons between two groups were performed using a paired Student’s *t* test. For comparison of more than two groups, one-way or two-way analysis of variance (ANOVA) was used with the recommended multiple comparison tests as indicated in the figure legend and as recommended by GraphPad Prism. Differences of *p* < 0.05 were considered significant.

## Results

### Clozapine reduces infiltration into the CNS

**Fig. 1:**
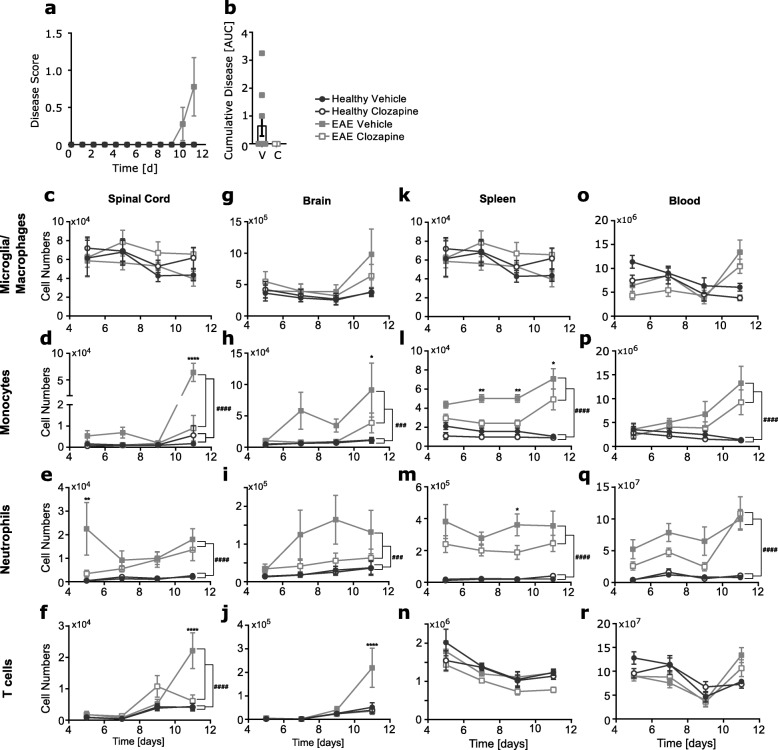
Clozapine treatment reduces infiltration into the CNS during the onset of EAE. C57BL/6 female mice were treated with clozapine (60 mg/kg/day) or vehicle control in their drinking water commencing one day prior to immunization and were scored daily (**a**, **b**). At day 5, 7, 9, and 11 after EAE induction spinal cord (**c**–**f**), brain (**g**–**j**), spleen (**k**–**n**), and blood (**o**–**r**) was collected and cell numbers were analyzed by flow cytometry. Shown are the means and SEM of individual mice (*n* = 9/treatment group) from three independent experiments normalized to healthy vehicle for each day. *p < 0.0332, **p < 0.021, and ***p < 0.0001 by two-way ANOVA with Tukey’s multiple comparisons test comparing EAE vehicle versus EAE Clozapine. ^###^*p* < 0.0002 and ^###^*p* < 0.0001 by three-way ANOVA comparing healthy versus EAE

Clozapine has been shown to reduce disease severity in the EAE model of MS when administered prophylactically or therapeutically [20, 21]; however, the mechanism of protection is not clear. To understand if clozapine altered the early infiltration of immune cells into the CNS post immunization, female C57BL/6 mice were treated with clozapine or vehicle commencing 1 day prior to immunization throughout the whole course of the experiment and the number and type of immune cells in the spinal cord and brain were analyzed by flow cytometry 5, 7, 9, and 11 days after EAE induction (Additional file 5: Figure S5a,b).). As expected, the onset of disease occurred 10 days after EAE induction in the vehicle-treated animals while clozapine-treated animals showed no overt signs of disease following EAE induction (Fig. 1a, b). Microglia numbers in the spinal cord and brain did not change over time or with different treatments (Fig. 1c, g). In the spinal cord, infiltrating neutrophils were observed at day 5 in EAE mice whereas clozapine treatment significantly reduced neutrophil cell numbers (Fig. 1e). Additionally, monocyte and neutrophil cell numbers were reduced in the spinal cords and brains of clozapine-treated EAE animals compared to vehicle over the whole time of observation (Fig. 1d, e, h, i). T cell infiltration was significantly reduced in clozapine-treated EAE mice compared to vehicle at day 11 in the spinal cord as well as in the brain (Fig. 1f, j). Together, these results indicate that clozapine reduces the infiltration of monocytes, neutrophils, and T cells pre and post disease onset.

To determine if the reduction in immune cell infiltration into the CNS correlated to immune cells in peripheral sites, similar assessments were done in whole blood and isolated splenocytes. Both monocytes and neutrophils numbers in the spleen and blood were elevated by EAE and were significantly reduced in the spleen in clozapine-treated EAE mice compared to vehicle (Fig. 1l, m). In contrast to the CNS, macrophage and T cell numbers were not altered by time, treatment, or immunization (Fig. 1k, n, o, r). Aside from a reduction in monocyte and neutrophil recruitment to the spleen by clozapine treatment, cell numbers of macrophages and T cells were not significantly altered in the spleen or blood pre and post EAE onset.

### Clozapine reduces the expression of CCL2 and CCL5 in the CNS

**Fig. 2:**
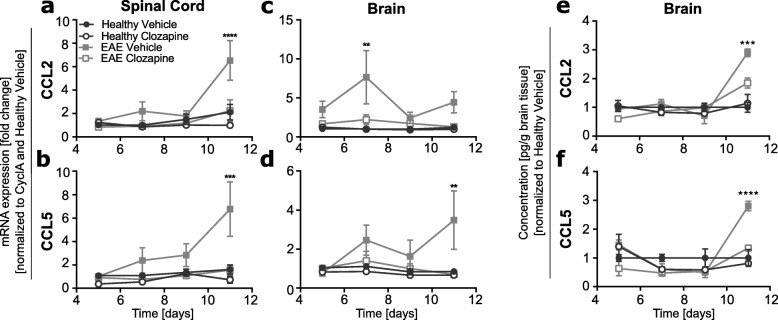
Clozapine treatment decreases mRNA and protein CCL2 and CCL5 expression in the CNS during the onset of EAE. C57BL/6 female mice were treated with clozapine or vehicle control in their drinking water. At day 5, 7, 9, and 11 after EAE induction spinal cord and brain was collected. **a**–**d** RNA was extracted and analyzed by qRT-PCR for CCL2 and CCL5 expression. Shown are the means and SEM of individual mice (*n* = 9/treatment group) from three independent experiments normalized to cyclophilin A as a housekeeper and healthy vehicle for each day. **e**, **f** Brains were lysed and protein expression was analyzed by Milliplex. Shown are the means and SEM of individual mice (*n* = 3/treatment group) normalized to healthy vehicle for each day. *p < 0.0332, **p < 0.021, ***p < 0.0002, and ****p < 0.0001 by two-way ANOVA with Tukey’s multiple comparisons test

Because these results indicate that upon clozapine treatment, less immune cells infiltrate into the CNS during the early period of disease onset, we investigated the expression of chemokines and cytokines in the CNS. Applying a 32-plex for the analysis of 32 different cytokines known to be involved in EAE [10, 25, 26], we found that only a few of the analyzed cytokines were regulated by EAE and clozapine treatment (Additional file 1: Figure S1e). Two chemokines, CCL2 and CCL5, which showed already an upregulation at the early timepoints were analyzed in more detail. These two cytokines are also known to recruit monocytes, T cells, and neutrophils to the sites of inflammation, and are known to be important in CNS inflammation during EAE [12, 13]. Analysis of CCL2 and CCL5 mRNA expression over time revealed that both transcripts increased in the spinal cords of vehicle-treated EAE compared to healthy animals by 11 days post-immunization and CCL2 and CCL5 levels were reduced by clozapine treatment (Fig. 2a,b). In the brain, CCL2 mRNA was increased at 7 days post-immunization and then returned to normal, while CCL5 mRNA was increased at 11 days post-immunization. As in the spinal cord, treatment with clozapine reduced the levels of chemokine expression to that of healthy control animals (Fig. 2c, d). The reduced chemokine expression of mRNA in the brain was also reflected in the expression of the protein where a significant difference between vehicle and clozapine-treated EAE animals was detected day 11 post EAE induction (Fig. 2e, f). Although EAE did significantly increase CCL2 expression in the blood 11 days post immunization, clozapine treatment did not alter it (Additional file 2: Figure S2c). No difference in CCL5 mRNA expression was detected in the blood (Additional file 2: Figure S2d) and for both chemokines in the spleen (Additional file 2: Figure S2a, b) of healthy and EAE mice treated either with vehicle or clozapine. Overall, the reduction in EAE-induced CCL2 and CCL5 in the spinal cord and brain but not spleen or blood parallels the reduction in immune cell recruitment into the CNS but not peripheral sites in clozapine-treated animals.

### Resident microglia and macrophages are targeted by clozapine for reduced chemokine expression

**Fig. 3:**
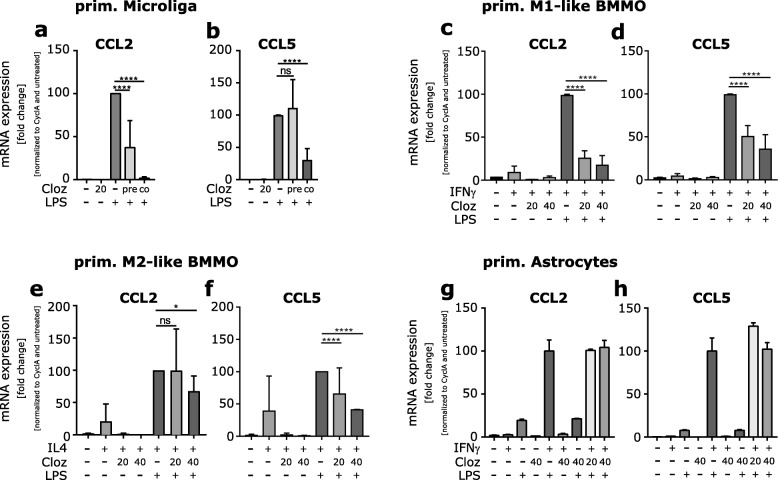
Clozapine treatment reduces CCL2 and CCL5 mRNA expression by primary macrophages and microglia. **a**, **b** Primary microglia were isolated from the brains of 5 days old mice, differentiated with M-CSF for 30 days and pre or co-treated with clozapine in the presence of LPS for 24 h. **c**–**f** Bone marrow-derived macrophages were isolated, differentiated with either GM-CSF and IL3 (**c**, **d**) or M-CSF (**e**, **f**) for 9 days and treated with clozapine in the presence of either IFNγ and LPS (**c**, **d**) or IL4 and LPS (**e**, **f**) for 24 h. **g**, **f** Primary astrocytes were isolated from brains and treated with clozapine in the presence of IFNγ and LPS for 24 h. For all samples, mRNA was extracted and analyzed by qRT-PCR for CCL2 and CCL5 expression. Shown are the means and SEM of three independent experiments normalized to cyclophilin A as a housekeeper and untreated control. *p < 0.0332 and ***p < 0.0001 by paired one-way ANOVA with Sidak’s multiple comparisons test

To test if clozapine reduces the expression of CCL2 and CCL5 in the CNS by directly targeting the resident microglia and to confirm which cells may be the source of these chemokines, primary microglia were derived as described previously [23] and astrocyte-rich cultures were isolated using the Neural Tissue Dissociation Kit (T), and activated by LPS in the presence or absence of clozapine. Upon activation of microglia, a high expression of CCL2 and CCL5 mRNA was detected (Fig. 3a, b). The expression of CCL2 was reduced if the microglia were pre- or co-treated with clozapine (Fig. 3a) but CCL5 was only significantly reduced by co-treatment (Fig. 3b). To investigate the effect of the activation status of macrophages and their role in cytokine secretion in EAE and in response to clozapine treatment, classically (M1-like) or alternatively (M2-like) activated bone marrow-derived macrophages were generated. Similar results were observed in these macrophages. Activation of these macrophages by either IFNγ and LPS or IL4 and LPS increased the expression of CCL2 and CCL5 mRNA while clozapine treatment reduced the expression (Fig. 3c–f). In contrast, while primary astrocytes showed an increased expression of CCL2 and CCL5 mRNA upon activation with LPS and IFNγ, treatment with clozapine had no effect on CCL2 or CCL5 expression at the time of observation (Fig. 3g, h). These findings indicate that microglia and macrophages present in the CNS at the time of disease induction may be directly targeted by clozapine leading to reduced activation of these cells [21], reduced chemokine expression, and consequently, changing infiltration into the CNS.

### Clozapine inhibits migration in the presence of excessive chemokines

**Fig. 4:**
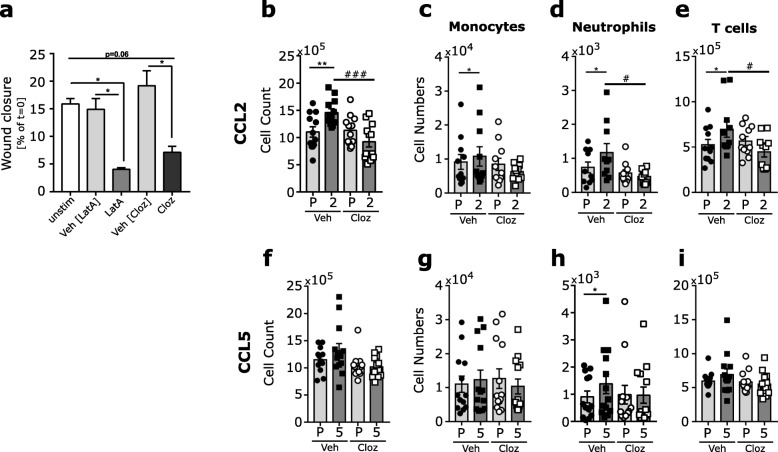
Clozapine treatment reduces CCL2- and CCL5-mediated migration. **a** In-vitro migration assay of bone marrow-derived macrophages treated with Latrunculin A or clozapine. **b**–**i** C57BL/6 female mice were treated with clozapine or vehicle control in their drinking water for 7 days. At the last day, CCL2 or CCL5 (left hind flank) or PBS (right hind flank) was injected s.c., and 18 h later, the draining LN cells were isolated and analyzed by flow cytometry. The total number of LN cells after CCL2 (**b**) or CCL5 injection (**f**) and of the individual cell types in the LN after CCL2 (**c**–**e**) or CCL5 injection (**g**–**i**) from three independent experiments (*n* = 13–14/group) are shown. P—PBS, 2—CCL2, 5—CCL5, *p < 0.0332 and **p < 0.021 by paired one-way ANOVA with Sidak’s multiple comparisons test within vehicle and clozapine treated groups. Comparison between CCL treatment in vehicle and clozapine mice was done by two-way ANOVA with Tukey’s multiple comparison test #*p* < 0.0332, ##*p* < 0.021, ###*p* < 0.0002, and ####*p* < 0.0001

To verify if the reduced migration into the CNS is due solely to reduced chemokine expression, or if migration is directly altered by clozapine, in-vitro migration experiments were performed. The Oris wound healing system was used wherein GM-CSF/IL3-derived bone marrow-derived macrophages were seeded in wells containing a plug. After settlement of the cells, the plug was removed to leave a “wound” or specific area free of cells. Macrophages were unstimulated or stimulated with clozapine, Latrunculin A, or respective vehicle. As shown in Fig. 4a and Additional file 3: Figure S3a, bone marrow-derived macrophages left untreated or treated with the respective vehicles migrate to close the wound. As expected, migration was significantly inhibited by Latrunculin A, which is known to inhibit migration by preventing actin polymerization. Interestingly, clozapine also significantly inhibited the migration of macrophages in this assay (Fig. 4a and Additional file 3: Figure S3a) showing that migration can be directly inhibited by clozapine without any other stimulatory factors.

To analyze if in-vivo chemokine-mediated migration is also directly inhibited by clozapine in the presence of excessive chemokine, an in-vivo migration assay was performed. To this end, CCL2 (abbreviated in Fig. 4 as “2”) or CCL5 (abbreviated in Fig. 4 as “5”) was injected s.c. in the lower left hind limb of mice, and PBS (abbreviated in Fig. 4 as “P”) was injected s.c. in the right lower hind limb as a control. Eighteen hours later, the draining lymph nodes were collected and analyzed by flow cytometry to enumerate and phenotype the immune cells (Additional file 6: Figure S6a). To determine the effect of clozapine, animals were treated with vehicle or clozapine for 7 days prior to administration of CCL2 or CCL5. Injection of CCL2 in vehicle-treated animals significantly increased cell numbers in the draining lymph nodes (Fig. 4b) compared to PBS injection, as expected. Interestingly, clozapine treatment significantly abolished this effect with similar cell numbers detected in the lymph nodes draining the site of CCL2 or PBS injection (Fig. 4b). Analysis of the cellular populations showed that monocytes, neutrophils, and T cells were significantly increased in the CCL2-affected lymph nodes of vehicle-treated mice, whereas the cellular populations in the lymph nodes were similar between PBS or CCL2 injection in clozapine-treated animals (Fig. 4c–e, Additional file 3: Figure S3b-f).

In contrast to CCL2, CCL5 injection did not induce a significant increase in total cells in the draining lymph nodes in the vehicle-treated animals and similar numbers were found in clozapine-treated animals (Fig. 4f). When the individual cellular populations were assessed, a significant increase in the number of neutrophils was detected in vehicle-treated mice injected with CCL5 (Fig. 1h) while other cell populations were constant (Fig. 4g, i and Additional file 3: Figure S3g-k). Additionally, the increase in neutrophils was not observed in clozapine-treated mice (Fig. 4h). This result reveals that neutrophils are the main cell type responding to CCL5 in this model (Fig. 4 h). Overall, these findings indicate that clozapine reduces chemokine-induced migration of immune cells in-vivo by directly targeting the migratory potential of the immune cells.

### Chemokine receptors are not altered by clozapine

**Fig. 5:**
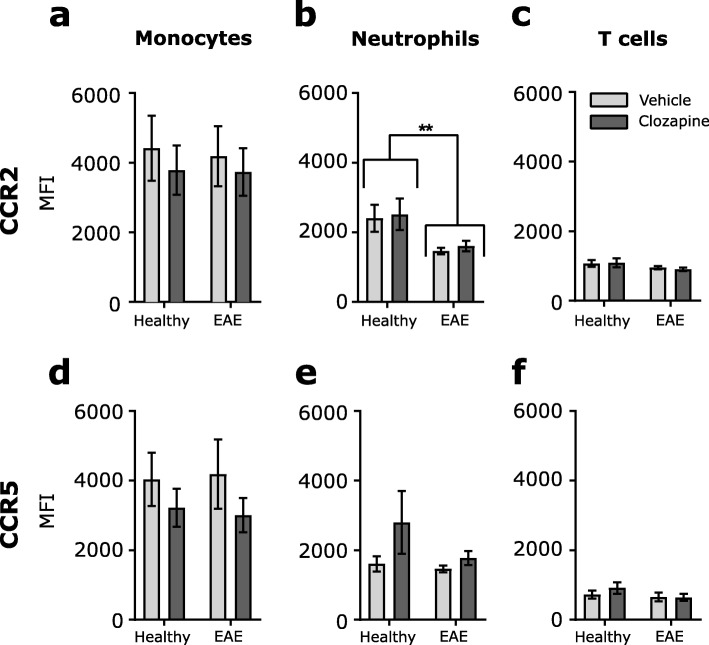
CCR2 and CCR5 expression is similar between vehicle and clozapine treatment. C57BL/6 female mice were treated with clozapine or vehicle control in their drinking water for 7 days, blood was collected and analyzed by flow cytometry for CCR2 (**a**–**c**) and CCR5 (**d–f**) expression. Shown are the MFI and SEM of individual mice (*n* = 13/treatment group).*p < 0.0332, **p < 0.021, ***p < 0.0002, and ****p < 0.0001 by one-way ANOVA with Sidak’s multiple comparisons test

To evaluate how clozapine reduces the chemokine-mediated migration, the expression of the chemokine receptors for CCL2 (CCR2) and CCL5 (CCR5) was analyzed on peripheral blood immune cells from healthy and EAE mice at day 7 after immunization treated with vehicle or clozapine commencing 1 day prior immunization; reflecting the first wave of infiltration into the CNS. Fig. 5 shows the expression of these two receptors on monocytes, neutrophils, and T cells (Fig. 5a–f) and reveals that overall, receptor expression was not altered in EAE animals compared to healthy control, except for a decrease in mean fluorescence intensity (MFI) for CCR2 on neutrophils in EAE compared to healthy control. However, no differences could be detected between vehicle or clozapine-treated mice following 7 days of EAE induction (Fig. 5). In addition, the frequency of CCR-positive cells within each cell type was also not altered by clozapine treatment (Additional file 4: Figure S4 a, b). From these results, we conclude that clozapine does not alter the expression of chemokine receptors directly to reduce migration but instead may alter factors downstream of these receptors.

### Clozapine inhibits migration by upregulation of cyclic AMP

**Fig. 6:**
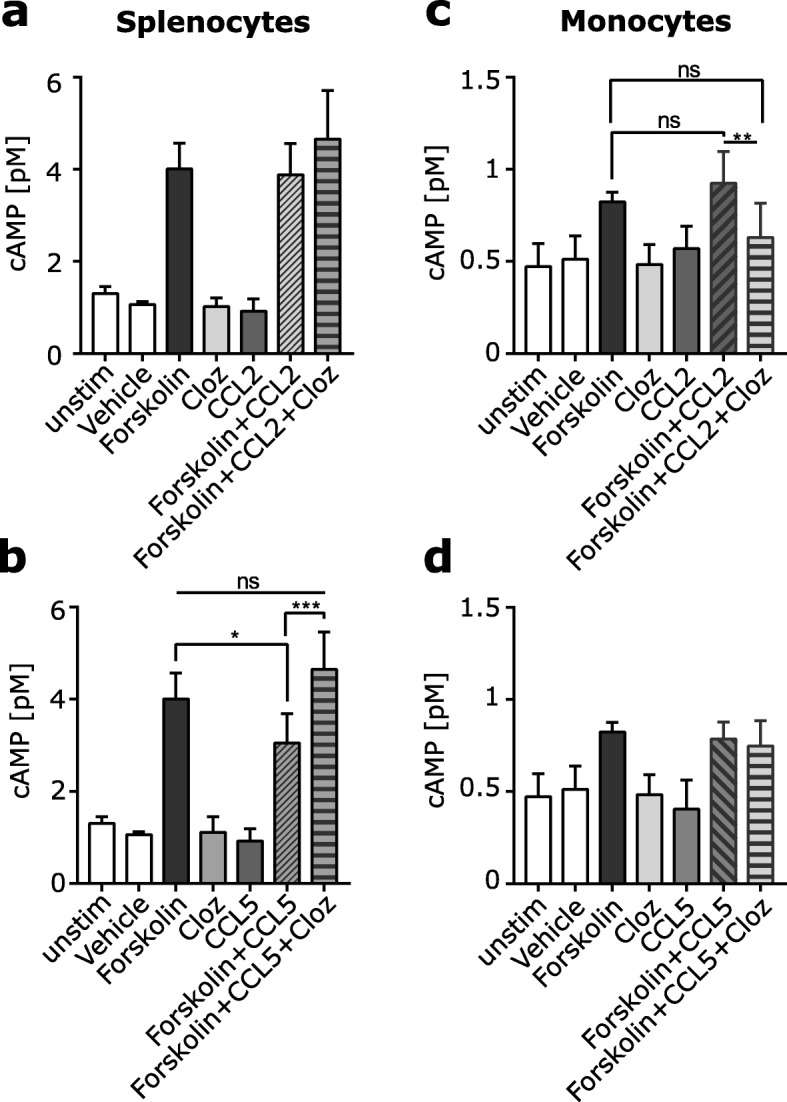
cAMP expression is altered by clozapine in splenocytes. Splenocytes (**a**, **b**) or sorted monocytes (**c**, **d**) were left untreated or stimulated with forskolin, CCL2 or CCL5 and clozapine in a single treatment or with different combinations and cAMP accumulation was measured with in the cells. Two technical replicates in three independent experiments per treatment group. *p < 0.0332, **p < 0.021, ***p < 0.0002 and by two-way ANOVA with Tukey’s multiple comparisons test

All chemokine receptors are part of a family of G-protein coupled receptors and one of the consequences of chemokine receptor activation is a change in cAMP within the cell. High cAMP concentrations are associated with reduced cellular locomotion, adhesion, and migration (Iwasaki 1994; Laudanna 1997; ParmoCabanas 2004). Therefore, we assessed if cAMP levels changed in different immune cell populations after CCL2 and CCL5 treatment in the presence or absence of clozapine. As expected, stimulation for 18 h with forskolin, a labdane diterpene, led to elevated intracellular cAMP in cultured splenocytes compared to the vehicle treatment (Fig. 6a, b). When CCL5 (Fig. 6b) was added with forskolin, a significant reduction in cAMP concentration was observed compared to forskolin alone suggesting that high chemokine concentrations reduced intracellular cAMP concentration, which would increase migration. The addition of clozapine to splenocyte cultures containing both forskolin and CCL5 significantly increased the intracellular cAMP levels (Fig. 6b) suggesting reduced migration. However, no significant effect was detected with the addition of clozapine treatment to forskolin and CCL2 (Fig. 6a).

Because > 90% of splencocytes from healthy mice are B or T cells and these cells express lower levels of CCR2 (Additional file 4: Figure S4c, e), we repeated these experiments using isolated monocytes, which are one of the main cell types expressing CCR2. Monocytes were isolated from the spleen and stimulated with forskolin, the chemokines, and clozapine. Additionally, monocytes were stimulated with LPS to further increase cAMP concentrations, as in preliminary experiments only low levels of cAMP were detected without LPS stimulation (Additional file 4: Figure S4h). The addition of LPS did not change the expression of CCR2 on the cell surface of cultured monocytes (Additional file 4: Figure S4d, f). Forskolin stimulation in monocytes led to only a minor increase in cAMP levels and addition of the chemokines CCL2 or CCL5 did not alter the cAMP levels (Fig. 6c, d). While treatment with clozapine in addition to forskolin and CCL5 did not show an effect (Fig. 6d), treatment with clozapine in addition to forskolin and CCL2 unexpectedly reduced the cAMP levels compared to forskolin treatment or in combination with CCL2 and was no different to forskolin treatment alone (Fig. 6c). Overall, we found that clozapine inhibited CCL5-mediated downregulation of cAMP in splenocytes but unexpectedly decreased cAMP levels in CCL2 and forskolin-treated monocytes, and together, these findings indicate that clozapine can directly alter CCL5 and CCL2-mediated signal pathways in immune cells.

## Discussion

The aim of this study was to investigate how the atypical antipsychotic agent, clozapine, reduces disease onset and severity in the EAE model of MS. EAE is characterized by a high infiltration of monocytes, neutrophils, and T cells into the CNS at the peak of disease [10, 25], and in this study, we found that clozapine reduced the infiltration of immune cells into the CNS and decreased the chemokine expression in the CNS in the early pre-symptomatic phase. In addition to reducing CCL2 and CCL5 expression, we found that clozapine treatment directly inhibited chemokine-mediated migration and signal pathways in immune cells. This study is the first to report a direct effect of clozapine on chemokine-induced signal pathways and reveals a novel mechanism by which clozapine may modulate immune responses.

In EAE, high chemokine production in the CNS is associated with acute disease symptoms [27], and chemokines, including MIP, CCL2, CCL3, CCL4, CCL5, CCL12, and CCL22, have been shown to play a role in development of EAE [28–30].

At the peak of disease, 14 days after immunization, high expression of the chemokines CCL2 and CCL5 were detected in the brain tissue of mice [13] and spinal cord [27]. Additionally, elevated CCL5 levels have been reported in the cerebrospinal fluid (CSF) of patients during MS relapses [31]. In contrast, CCL3 expression was not significantly changed at disease onset, although it is possible that it may play a role in later phase of EAE [13]. Here, the analysis of the early regulation of inflammatory chemokines showed that already by 7 days after immunization, an enhanced expression of CCL2 and CCL5 could be detected in brain tissue with the expression increasing by 11 days post-immunization in the spinal cord. This finding is in line with Borjini et al. where they showed increased chemokine expression in the onset phase of EAE in a rat model of multiple sclerosis. CCL2 was highly present in the cerebrospinal fluid (CSF) 8 days after EAE induction, while CCL5 was highest at 11 and 18 days after EAE induction [26]. This early expression of chemokine genes was also detected in the spinal cord 4 days after adoptively transferred T cells in the passive induction model of murine EAE [32]. In contrast to other chemokines of the C-C family, which trigger the Th1 phenotype upon their interaction with CCR5 on T-helper cells [33], CCL2 expression is associated with the polarization of Th0 cells toward a Th2 phenotype [34, 35]. This is characterized by an enhanced of IL-4 by T cells induced by CCL2 [36] and this early expression of CCL2 may also be associated with initiation of the blood-brain barrier breakdown leading to enhanced infiltration.

To investigate which cell types are the major source for CCL2 and CCL5 expression in the CNS and which cell types are targeted by clozapine treatment, primary microglia and astrocytes were isolated and bone marrow-derived macrophages were generated and treated with clozapine after activation. It has been shown that CCL2 is expressed by astrocytes and macrophages within actively demyelinating MS plaques [37] and astrocytes are known to be the likely cellular source of CCL2 in MS and EAE [38, 39]. Our results are in agreement with the previous published data showing that CCL2 and CCL5 are highly expressed after astrocyte activation; however, treatment with clozapine did not result in a decreased expression in astrocytes. However, CCL2 expression by astrocytes has been shown to be critical for the ongoing inflammation in chronic EAE and not for the induction phase [40]. Thus, CCL2 expression on other CNS resident cells may be important in the initial early inflammatory processes at disease onset.

Another major source of chemokine expression within the CNS is resident activated microglia. The production of CCL2 from resident microglia contributes to the recruitment of leukocytes into the CNS in EAE [41]. In our study, we could show that CCL2 and CCL5 mRNA was highly expressed after microglia activation and that either clozapine pre-treatment or co-treatment reduced the LPS-induced expression of CCL2 and CCLL5 mRNA. This reduction is likely due to reduced activation of microglia by clozapine as it has been shown previously by our group that clozapine reduces the expression of the activation markers I-A and CD40 on microglia in the brain and spinal cord during EAE [21]. In addition, clozapine was also shown to inhibit microglia activation in culture as measured by the reduced expression of Iba1 [42], and clozapine pre-treatment resulted in the suppression of LPS-induced expression of IL-1β, IL-6, and iNOS mRNA in both BV2 and primary cultured rat microglial cells [43]. We also found that bone marrow-derived macrophages are capable of expressing CCL2 and CCL5 after activation and that this expression was reduced with clozapine treatment. While in our case, it did not make a difference whether the macrophages were derived using M-CSF or GM-CSF, it has been shown previously that M-CSF-derived macrophages exhibit higher CCL2-dependent monocyte recruitment than GM-CSF-derived, whereas only the latter are capable of responding to CCL2 [44]. Overall, we show that microglia and macrophages are a major target of clozapine treatment leading to the reduced expression of the chemoattractant cytokines CCL2 and CCL5.

In addition to demonstrating a reduction in the signals driving migration into the CNS during EAE, we also found that clozapine directly inhibits the ability of these immune cells to migrate. Using an in-vitro migration assay of wound healing and an in-vivo CCL2 or CCL5-driven migration assay, we showed for the first time that migration was inhibited in the presence of clozapine. Our results suggest that clozapine may directly target the migrating immune cells and prevent their response to chemotactic signals like CCL2 and CCL5. The chemokine receptors CCR2 and CCR5 have been shown to be critically important in controlling leukocyte migration across endothelium and the blood-brain barrier [45] and to play an important role in the development and severity of EAE. CCR2^−/−^ mice immunized with MOGp35–55 failed to develop mononuclear cell inflammatory infiltrates in the CNS and failed to increase CNS levels of the chemokines, MCP-1, and interferon (IFN)-inducible protein 10 (IP-10) as well as CCR1, CCR2, and CCR5 [46]. Additionally, deficiency in CCR5 suppresses EAE in C57BL/6 mice by reduced immune cells infiltration and astrocyte and microglia activation [47]. However, our work revealed that the block in migration was not mediated by changes in the expression of CCR2 and CCR5 on immune cells in the blood of EAE animals where we saw an overall reduction of CCR2 and CCR5 on neutrophils compared to healthy animals, but no difference with clozapine treatment. However, it was shown that CCR5 is present only on a small subset of circulating blood monocytes, but is highly increased on all monocytes in MS lesions [48]. In line with this, Trebst et al. showed that monocytes showed higher expression of CCR5 in the CNS than in the blood [49]. There is a good correlation between high expression of CCR2 and CCR5 in the spinal cord and disease severity and the main cellular source of CCR1, CCR2, and CCR5 high expression were migrating inflammatory cells [15, 45, 47]. So immune cells expressing higher levels of CCR2 or CCR5 in the blood could be migrating through the BBB into the CNS and accumulating in the lesions. This would explain why a reduction of CCR2 and CCR5 on immune cells in the blood are detected in our study. In addition, high levels present of the corresponding chemokines might lead to a faster and more frequent internalization of the existing chemokine receptors on the cell surface. Once the chemokine binds to its corresponding receptor, they are quickly internalized, and the downstream signaling is activated. CCR2 has a half-life on the cell surface of around 30 min, while CCR5 is internalized after around 60 min [50] but depending on the ligand, affinity, and cell type. This might, in addition, explain why in EAE where high levels of the chemokines are present, the receptors are downregulated.

Given that the chemokine receptors were not targeted by clozapine treatment, we speculated that the block in the response to chemotactic signals may be due to an alteration in the signaling cascade downstream of the receptors by clozapine. One signaling molecule that is associated with migration is the second messenger cAMP. cAMP accumulation is a representative read out for CCR5 activation, where all CCR5 agonists inhibited the cAMP production triggered by forskolin [51]. Treatment with clozapine in addition to forskolin and CCL5 reversed the CCL5-induced downregulation of cAMP. In contrast, only a minor difference was seen with CCL2 co-treatment and in addition with clozapine. This lack of response may be due to the higher expression of CCR5 on lymphocytes than CCR2. While monocytes express high levels of CCR2, we were unable to demonstrate a reproducible effect of clozapine on cAMP levels in monocytes. cAMP has many different functions in different cell types; it has the ability to inhibit proliferation in some cell types while stimulating proliferation in others. Thus, cAMP has cell type-specific effects demonstrating that the same signaling molecule can have opposing effects in the different cells [52]. In activated macrophages, cAMP plays a key role in the regulation of an inflammatory response, and an increase in cAMP reduces cytokine and chemokine production by activated macrophages leading to a dramatical dampening of the inflammatory response [53]. Inhibition of migration and locomotion of cells is usually associated with an elevation of intracellular cAMP; however, this is also concentration-dependent and cell type-specific, especially in epidermal cell migration [54]. For example, the presence of cAMP can reduce the number of cells bound to the adhesion molecule VCAM1, and PKA inhibitors counteract the decreased adhesion to VCAM1 induced by cAMP [55] suggesting that cAMP-dependent PKA acts as a negative modulator on the chemoattractant [56].

Forskolin is a highly potent and rapid inducer of cAMP. In line with our data, studies have shown that different chemokines exhibited the capacity to inhibit forskolin-induced cAMP accumulation in a dose-dependent manner. CCL5 but not CCL2 treatment reduced the forskolin-stimulated cAMP formation in a dose-dependent manner in HEK293 cells [57], and CCL5 also inhibited adenylyl cyclase activity in cells transiently transfected with CCR1 [58]. The reports on CCL2 by different groups are more complicated. While Wang et al. and O’Boyle et al. showed that interaction of CCL2 with CCR2 causes cAMP inhibition and a significant reduction in the concentration of cAMP [59, 60], Mizutani et al. showed that CCL2 increases cAMP accumulation and that the differences of CCL2-induced cAMP accumulation may indeed dependent on cell type [61]. It has also been shown that different ligands for the same receptor resulted in different outcomes. CCL2 and CCL8 revealed different maximal inhibition of forskolin-induced cAMP production by both chemokines suggesting that CCR2-mediated cAMP alteration is concentration dependent [62]. Taken together, these finding may explain why there is a difference between the response of lymphocyte and monocytes to CCL5 and CCL2. Finally, cAMP is not the only signaling molecule activated downstream of the chemokine receptors CCR5 and CCR2. It is known that CCL2 treatment increases Ca^2+^ flux and leads to PI3k-Akt activation [59, 61]. All these different pathways may influence the migratory potential of cells with each pathway having a different influence in different cell types and future work is needed to elaborate the effect of signaling pathway activation in the presence of clozapine.

## Conclusion

In summary, this study is the first report of a novel mechanism of action for the atypical antipsychotic drug, clozapine, in EAE. Overall, we have shown that clozapine inhibited the migration of immune cells into the CNS in the EAE model of MS. This migration was prevented firstly by reduced chemokine expression in the CNS, and secondly by directly targeting the migratory potential of the cells. Out data suggests that clozapine did not alter the expression of the receptors directly but instead targeted the downstream signaling pathways that enable migration. Further studies need to elucidate in more detail the precise signaling pathways that are targeted and whether these activities are cell type-specific. Together, this work highlights a novel mechanism by which clozapine may exert the reported immunomodulatory effects that contribute to its known effectiveness in treating a wide range of neuroglial disorders including schizophrenia and Parkinson’s disease.

## Supplementary information

**Additional file 1: Figure S1.** Clozapine treatment reduces disease severity at the onset of EAE. C57BL/6 female mice were treated with clozapine (60 mg/kg/day) or vehicle control in their drinking water commencing one day prior to immunization and were scored and weight daily. At day 5, 7, 9 and 11 after EAE induction spinal cord (a), brain (b), spleen (c) and blood (d) was collected, cells were isolated and counted. Shown are the means and SEM of individual mice (*n* = 9/treatment group) from three independent experiments normalized to healthy vehicle for each day. (e) C57BL/6 female mice were treated with clozapine or vehicle control in their drinking water. At day 5, 7, 9 and 11 after EAE induction brain was collected, lysed and protein expression was analyzed by Milliplex. Shown are the means and SEM of individual mice (*n* = 3/ treatment group) normalized to healthy vehicle for each day.

**Additional file 2:Figure S2.** Clozapine treatment shows a minor effect on the cell numbers and CCL2 and CCL5 expression in spleen or blood. C57BL/6 female mice were treated with clozapine (60 mg/kg/day) or vehicle control in their drinking water commencing one day prior to immunization and were scored daily. At day 5, 7, 9 and 11 after EAE induction spleen (a,b) and blood (c,d) was collected and RNA was extracted and analyzed by qRT-PCR for CCL2 and CCL5 expression. Shown are the means and SEM of individual mice (n = 9/ treatment group) from 3 independent experiments normalized to cyclophilin A as a housekeeper and healthy vehicle for each day.

**Additional file 3: Figure S3.** Clozapine treatment reduces CCL2- and CCL5-mediated migration. (a) Representative images of BMMO in the in-vitro migration assay (b-k) C57BL/6 female mice were treated with clozapine (60 mg/kg/day) or vehicle control in their drinking water for 7 days. At the last day, CCL2 or CCL5 (left hind flank) or PBS (right hind flank) was injected s.c., and 18 h later, the draining LN cells were isolated and analyzed by flow cytometry. The total number of the individual cell types after CCL2 or CCL5 injection in the LN from 3 independent experiments (*n* = 13–14/group) are shown. P – PBS, 2 – CCL2, 5 – CCL5, *p < 0.0332 by paired 1-way ANOVA with Sidak’s multiple comparisons test.

**Additional file 4: Figure S4.** CCR2 and CCR5 expression is unaltered. C57BL/6 female mice were treated with clozapine (60 mg/kg/day) or vehicle control in their drinking water commencing one day prior to immunization and were scored daily. At day 5, 7, 9 and 11 after EAE induction blood was collected analyzed by flow cytometry for CCR2 (a) or CCR5 (b) expression. Shown are the frequency of the parent population and SEM of individual mice (n = 13/ treatment group). *p < 0.0332, **p < 0.021 and ***p < 0.0001 by 1-way ANOVA with Sidak’s multiple comparisons test. CCR2 and CCR5 expression on splenocytes (c,e) and sorted monocyte (d,f) after culture, representative flow plots and dMFI of receptor to isotype control. (g) cAMP measurement in splenocytes treated with forskolin and ConA. (h) cAMP measurement in sorted monocytes treated with forskolin and LPS. p < 0.0332, **p < 0.021 and by 2-way ANOVA with Tukey’s multiple comparisons test.

**Additional file 5:Figure S5.** Gating strategy for infiltration and in-vivo migration experiments. Gating strategy is shown for the (a) infiltration experiments for brain/spinal cord and spleen/blood (Fig. 1 c-r) and (b) in-vivo migration experiment for lymph nodes (Fig. 4 b-i and Additional file 3 b-k) from one EAE vehicle treated animal as an example, the same flow cytometry markers are used for both experiments: CD4-BV521 (RM4–5), CD45-BV510 (30-F11), CD25-AF488 (PC61), CD8-PerCPCy5.5 (53–6.7), CD11b-PE-Cy7 (M1/70), CD3-APC-Cy7 (17A2), Ly6C-PE (HK1.4), Ly6G-APC (1A8).

**Additional file 6:Figure S6.** Gating strategy for CCR expression in blood. Gating strategy is shown for CCR expression analysis in blood (Fig. 5 and Additional file 4 a,b) from one EAE vehicle treated animal as an example, the following antibodies were used to detect the populations: CD4-BV521 (RM4–5), CD45-BV510 (30-F11), CD8-PerCPCy5.5 (53–6.7), CD11b-PE-Cy7 (M1/70), CD45R-AF488 (RA3-6B2), CCR2-PE (475301), CCR5-APC (HM-CCR5) Gr1-APC-Cy7 (RB6-8C5).

## Data Availability

The datasets used and/or analyzed during the current study are available from the corresponding author on reasonable request.

## Abbreviations

AC: Adenylyl cyclases
Akt: Protein kinase B
CCL: Chemokine (C-C motif) ligand
CCR: C-C chemokine receptor
CNS: Central nervous systems
CSF: Cerebrospinal fluid
EAE: Experimental autoimmune encephalomyelitis
ERK: Extracellular signal-regulated kinases
GM-CSF: Granulocyte-macrophage colony-stimulating factor
IFNγ: Interferon gamma
IL: Interleukin
MCP-1: Monocyte chemoattractant protein 1
M-CSF: Macrophage colony-stimulating factor
MFI: Mean fluorescence intensity
MS: Multiple sclerosis
NOS: Nitric oxide synthases
PBS: Phosphate-buffered saline
PDE: Phosphodiesterases
PI3K: Phosphatidylinositol-4,5-bisphosphate 3-kinase
RANTES: Regulated upon activation normal T cell expressed and secreted
TNFα: Tumor necrosis factor alpha
VEGF: Vascular endothelial growth factor
