# Supplementary material for: Clozapine reduces infiltration into the CNS by targeting migration in experimental autoimmune encephalomyelitis
Source: J Neuroinflammation. 2020 Feb 12;17:53. doi: 10.1186/s12974-020-01733-4 (PMC7014621; doi:10.1186/s12974-020-01733-4)
Supplement: Supplementary file 1 — Additional file 1: Figure S1. Clozapine treatment reduces disease severity at the onset of EAE. C57BL/6 female mice were treated with clozapine (60 mg/kg/day) or vehicle control in their drinking water commencing one day prior to immunization and were scored and weight daily. At day 5, 7, 9 and 11 after EAE induction spinal cord (a), brain (b), spleen (c) and blood (d) was collected, cells were isolated and counted. Shown are the means and SEM of individual mice (n = 9/treatment group) from three independent experiments normalized to healthy vehicle for each day. (e) C57BL/6 female mice were treated with clozapine or vehicle control in their drinking water. At day 5, 7, 9 and 11 after EAE induction brain was collected, lysed and protein expression was analyzed by Milliplex. Shown are the means and SEM of individual mice (n = 3/ treatment group) normalized to healthy vehicle for each day. [file 12974_2020_1733_MOESM1_ESM.pdf]

Supplement Figure 1

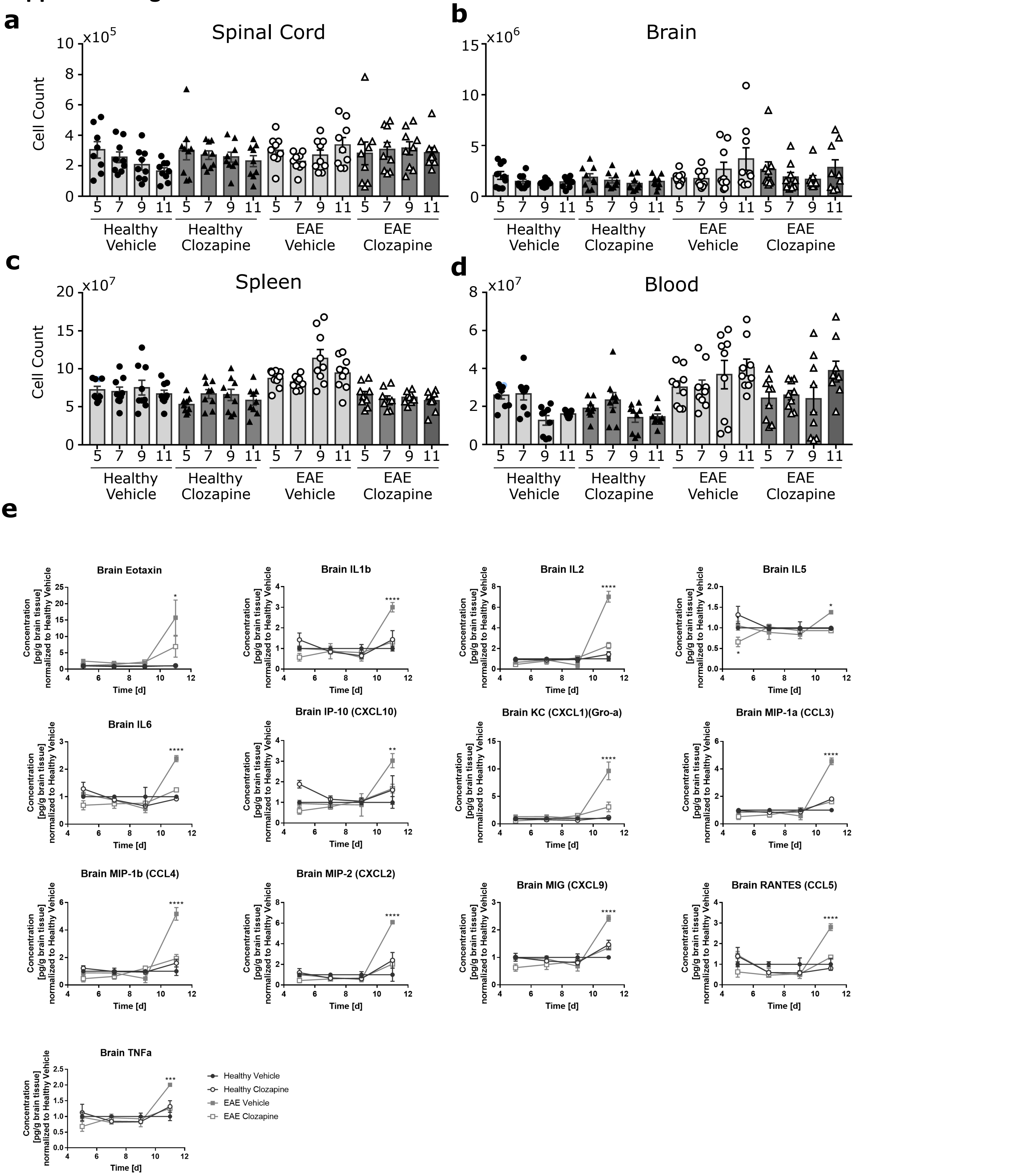

Additional file 1. Clozapine treatment reduces disease severity at the on-set of EAE. C57BL/6 female mice were treated with clozapine (60 mg/kg/day) or vehicle control in their drinking water commencing one day prior to immunization and were scored and weight daily. At day 5, 7, 9 and 11 after EAE induction spinal cord (a), brain (b), spleen (c) and blood (d) was collected, cells were isolated and counted. Shown are the means and SEM of individual mice (n = 9/treatment group) from three independent experiments normalized to healthy vehicle for each day. (e) C57BL/6 female mice were treated with clozapine or vehicle control in their drinking water. At day 5, 7, 9 and 11 after EAE induction brain was collected, lysed and protein expression was analysed by Milliplex. Shown are the means and SEM of individual mice (n = 3/ treatment group) normalized to healthy vehicle for each day.
