# Supplementary material for: Clozapine reduces infiltration into the CNS by targeting migration in experimental autoimmune encephalomyelitis
Source: J Neuroinflammation. 2020 Feb 12;17:53. doi: 10.1186/s12974-020-01733-4 (PMC7014621; doi:10.1186/s12974-020-01733-4)
Supplement: Supplementary file 2 — Additional file 2:Figure S2. Clozapine treatment shows a minor effect on the cell numbers and CCL2 and CCL5 expression in spleen or blood. C57BL/6 female mice were treated with clozapine (60 mg/kg/day) or vehicle control in their drinking water commencing one day prior to immunization and were scored daily. At day 5, 7, 9 and 11 after EAE induction spleen (a,b) and blood (c,d) was collected and RNA was extracted and analyzed by qRT-PCR for CCL2 and CCL5 expression. Shown are the means and SEM of individual mice (n = 9/ treatment group) from 3 independent experiments normalized to cyclophilin A as a housekeeper and healthy vehicle for each day. [file 12974_2020_1733_MOESM2_ESM.pdf]

Supplement Figure 2

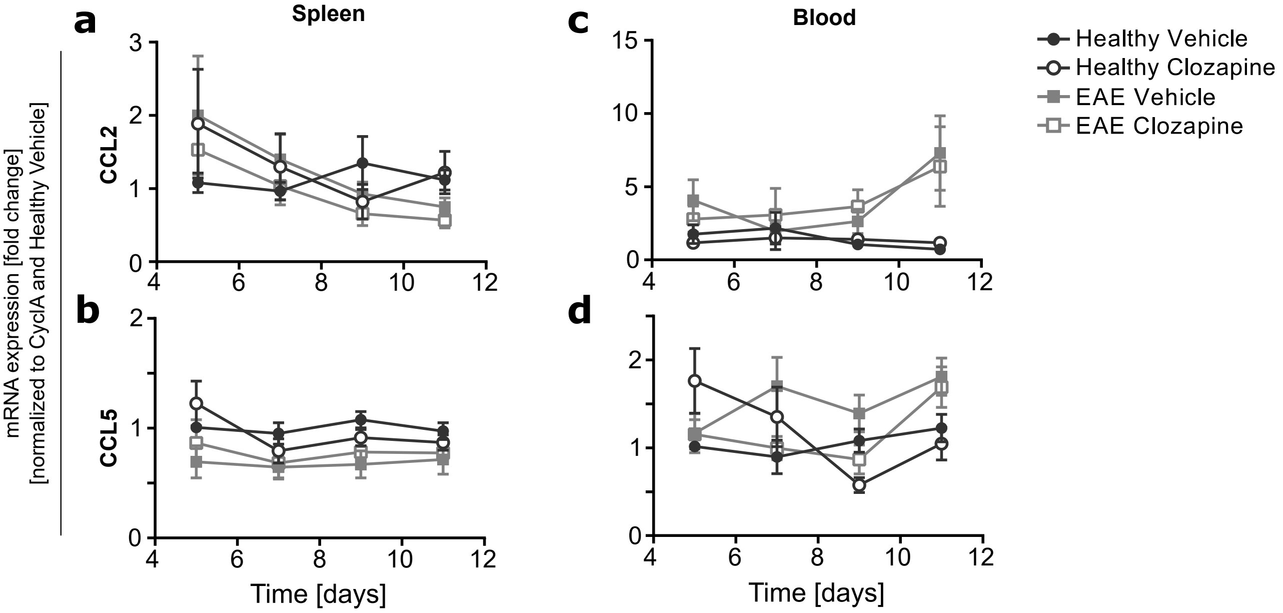

Additional file 2. Clozapine treatment shows a minor effect on the cell numbers and CCL2 and CCL5 expression in spleen or blood. C57BL/6 female mice were treated with clozapine (60 mg/kg/day) or vehicle control in their drinking water commencing one day prior to immunization and were scored daily. At day 5, 7, 9 and 11 after EAE induction spleen (a,b) and blood (c,d) was collected and RNA was extracted and analysed by qRT-PCR for CCL2 and CCL5 expression. Shown are the means and SEM of individual mice (n = 9/ treatment group) from 3 independent experiments normalized to cyclophilin A as a housekeeper and healthy vehicle for each day.
