# Supplementary material for: Clozapine reduces infiltration into the CNS by targeting migration in experimental autoimmune encephalomyelitis
Source: J Neuroinflammation. 2020 Feb 12;17:53. doi: 10.1186/s12974-020-01733-4 (PMC7014621; doi:10.1186/s12974-020-01733-4)
Supplement: Supplementary file 3 — Additional file 3: Figure S3. Clozapine treatment reduces CCL2- and CCL5-mediated migration. (a) Representative images of BMMO in the in-vitro migration assay (b-k) C57BL/6 female mice were treated with clozapine (60 mg/kg/day) or vehicle control in their drinking water for 7 days. At the last day, CCL2 or CCL5 (left hind flank) or PBS (right hind flank) was injected s.c., and 18 h later, the draining LN cells were isolated and analyzed by flow cytometry. The total number of the individual cell types after CCL2 or CCL5 injection in the LN from 3 independent experiments (n = 13–14/group) are shown. P – PBS, 2 – CCL2, 5 – CCL5, *p < 0.0332 by paired 1-way ANOVA with Sidak’s multiple comparisons test. [file 12974_2020_1733_MOESM3_ESM.pdf]

Supplement Figure 3

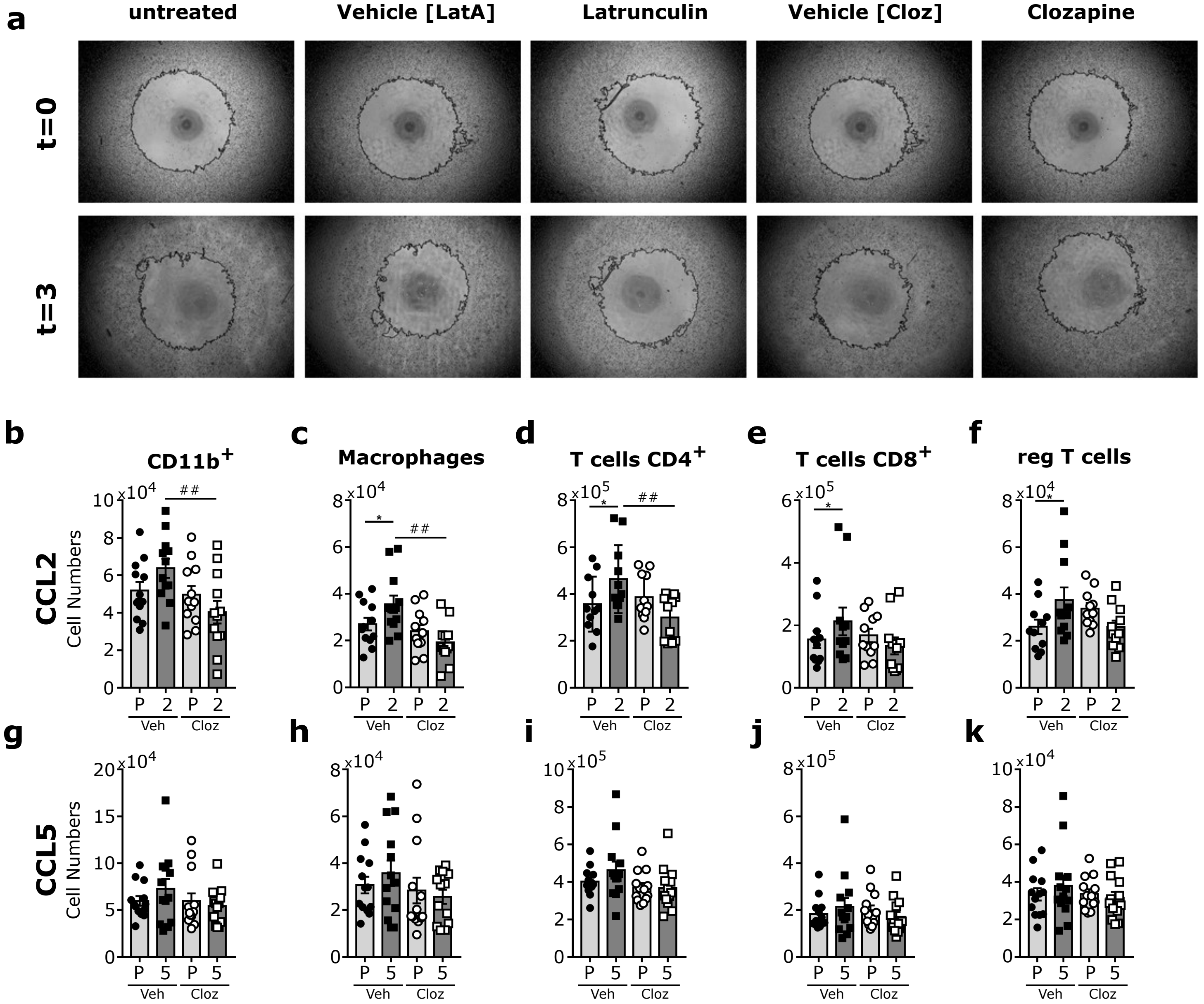

Additional file 3. Clozapine treatment reduces CCL2- and CCL5-mediated migration. (a) Representative images of BMMO in the in-vitro migration assay (b-k) C57BL/6 female mice were treated with clozapine (60 mg/kg/day) or vehicle control in their drinking water for 7 days. At the last day, CCL2 or CCL5 (left hind flank) or PBS (right hind flank) was injected s.c., and 18h later, the draining LN cells were isolated and analysed by flow cytometry. The total number of the individual cell types after CCL2 or CCL5 injection in the LN from 3 independent experiments (n=13-14/group) are shown. P – PBS, 2 – CCL2, 5 – CCL5, *p < 0.0332 by paired 1-way ANOVA with Sidak's multiple comparisons test.
