# Supplementary material for: Clozapine reduces infiltration into the CNS by targeting migration in experimental autoimmune encephalomyelitis
Source: J Neuroinflammation. 2020 Feb 12;17:53. doi: 10.1186/s12974-020-01733-4 (PMC7014621; doi:10.1186/s12974-020-01733-4)
Supplement: Supplementary file 4 — Additional file 4: Figure S4. CCR2 and CCR5 expression is unaltered. C57BL/6 female mice were treated with clozapine (60 mg/kg/day) or vehicle control in their drinking water commencing one day prior to immunization and were scored daily. At day 5, 7, 9 and 11 after EAE induction blood was collected analyzed by flow cytometry for CCR2 (a) or CCR5 (b) expression. Shown are the frequency of the parent population and SEM of individual mice (n = 13/ treatment group). *p < 0.0332, **p < 0.021 and ***p < 0.0001 by 1-way ANOVA with Sidak’s multiple comparisons test. CCR2 and CCR5 expression on splenocytes (c,e) and sorted monocyte (d,f) after culture, representative flow plots and dMFI of receptor to isotype control. (g) cAMP measurement in splenocytes treated with forskolin and ConA. (h) cAMP measurement in sorted monocytes treated with forskolin and LPS. p < 0.0332, **p < 0.021 and by 2-way ANOVA with Tukey’s multiple comparisons test. [file 12974_2020_1733_MOESM4_ESM.pdf]

Supplement Figure 4

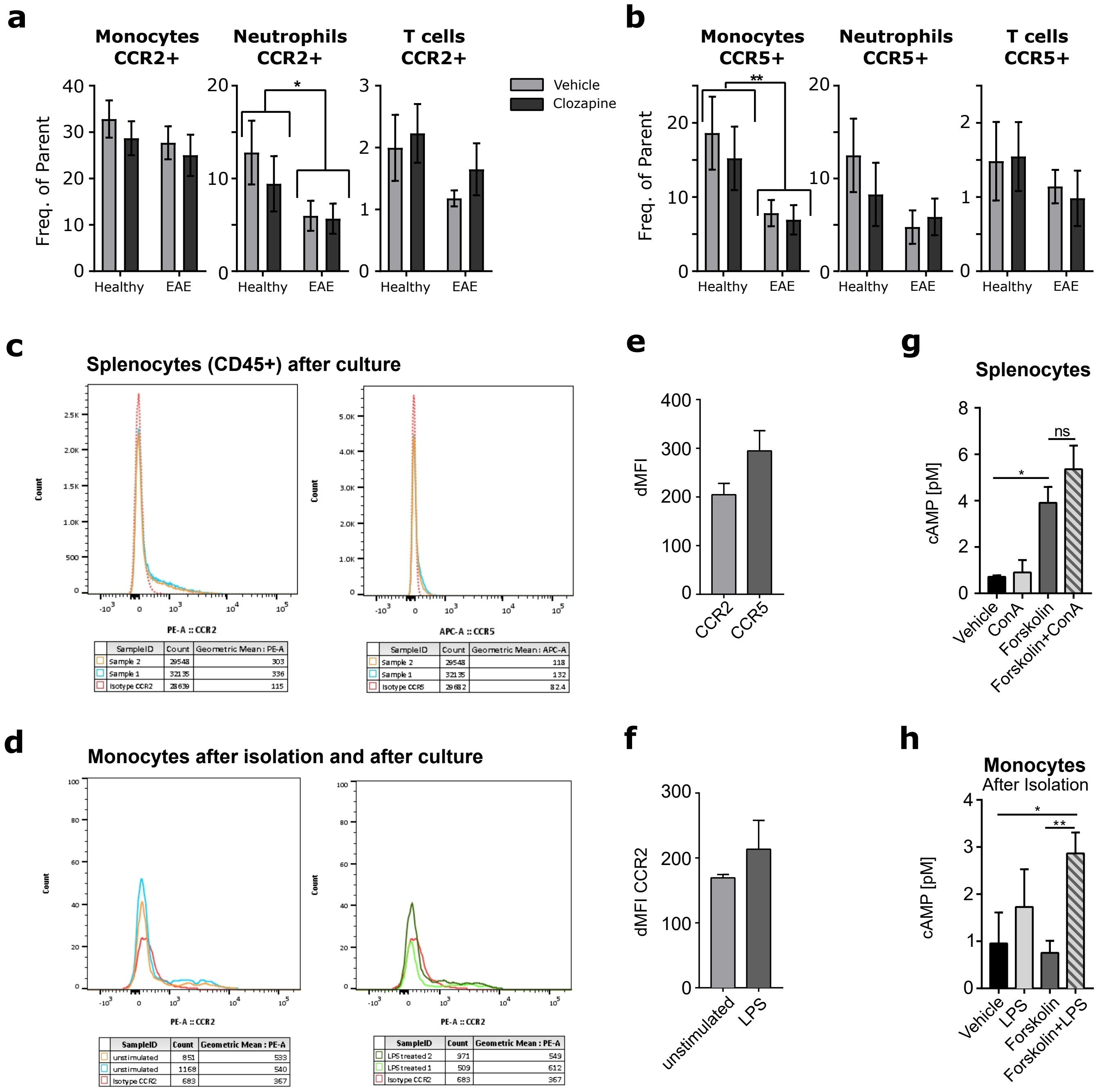

Additional file 4. CCR2 and CCR5 expression is unaltered. C57BL/6 female mice were treated with clozapine (60 mg/kg/day) or vehicle control in their drinking water commencing one day prior to immunization and were scored daily. At day 5, 7, 9 and 11 after EAE induction blood was collected analysed by flow cytometry for CCR2 (a) or CCR5 (b) expression. Shown are the frequency of the parent population and SEM of individual mice (n = 13/ treatment group). *$p < 0.0332$, **$p < 0.021$ and ***$p < 0.0001$ by 1-way ANOVA with Sidak's multiple comparisons test. CCR2 and CCR5 expression on splenocytes (c,e) and sorted monocyte (d,f) after culture, representative flow plots and dMFI of receptor to isotype control. (g) cAMP measurement in splenocytes treated with forskolin and ConA. (h) cAMP measurement in sorted monocytes treated with forskolin and LPS. $p < 0.0332$, **$p < 0.021$ and by 2-way ANOVA with Tukey's multiple comparisons test.
