# Supplementary material for: Clozapine reduces infiltration into the CNS by targeting migration in experimental autoimmune encephalomyelitis
Source: J Neuroinflammation. 2020 Feb 12;17:53. doi: 10.1186/s12974-020-01733-4 (PMC7014621; doi:10.1186/s12974-020-01733-4)
Supplement: Supplementary file 5 — Additional file 5:Figure S5. Gating strategy for infiltration and in-vivo migration experiments. Gating strategy is shown for the (a) infiltration experiments for brain/spinal cord and spleen/blood (Fig. 1 c-r) and (b) in-vivo migration experiment for lymph nodes (Fig. 4 b-i and Additional file 3 b-k) from one EAE vehicle treated animal as an example, the same flow cytometry markers are used for both experiments: CD4-BV521 (RM4–5), CD45-BV510 (30-F11), CD25-AF488 (PC61), CD8-PerCPCy5.5 (53–6.7), CD11b-PE-Cy7 (M1/70), CD3-APC-Cy7 (17A2), Ly6C-PE (HK1.4), Ly6G-APC (1A8). [file 12974_2020_1733_MOESM5_ESM.pdf]

Supplement Figure 5

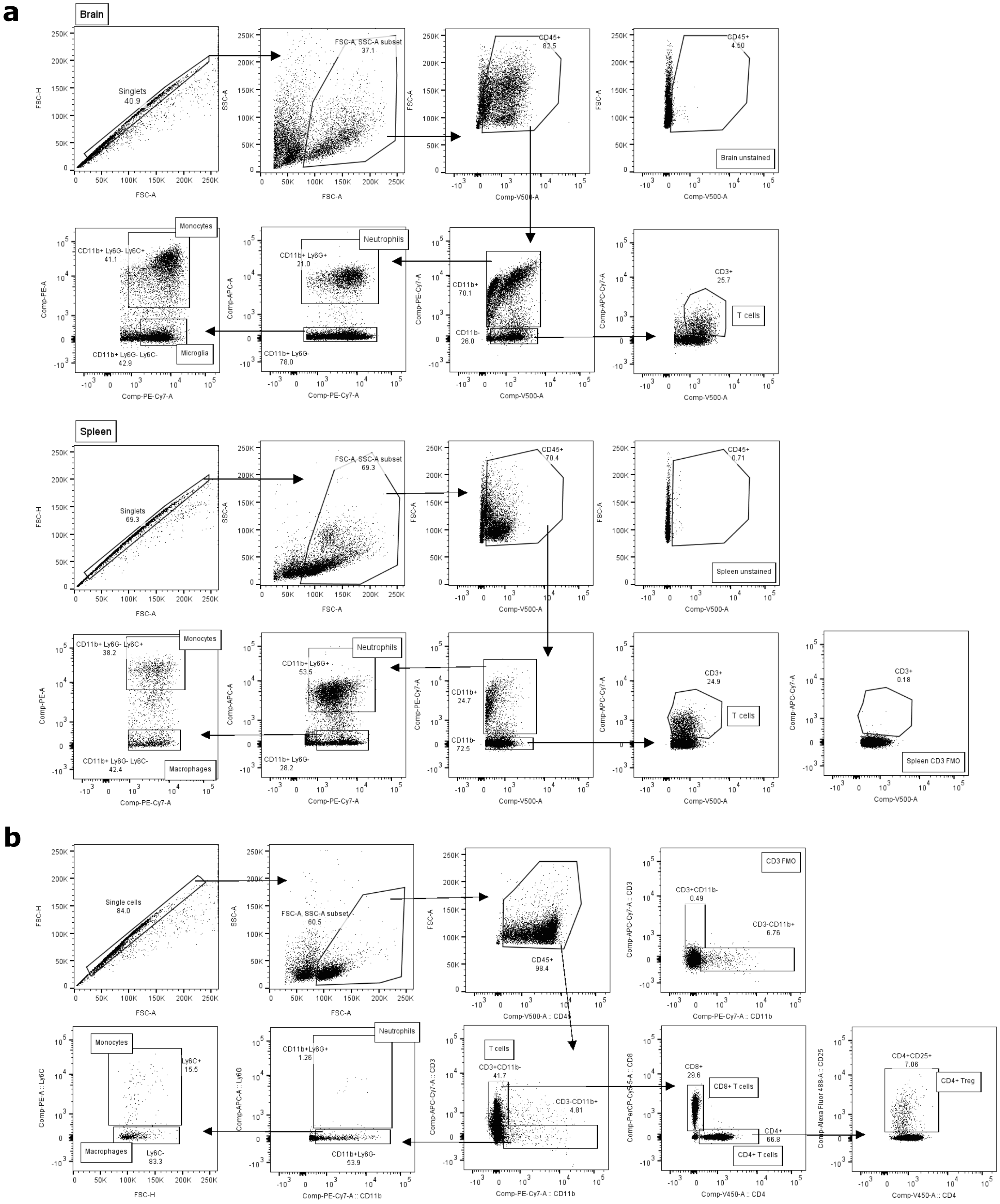

Additional file 5. Gating strategy for infiltration and in-vivo migration experiments. Gating strategy is shown for the (a) infiltration experiments for brain/spinal cord and spleen/blood (Figure 1 c-r) and (b) in-vivo migration experiment for lymph nodes (Figure 4 b-i and Additional file 3 b-k) from one EAE vehicle treated animal as an example, the same flow cytometry markers are used for both experiments: CD4-BV521 (RM4-5), CD45-BV510 (30-F11), CD25-AF488 (PC61), CD8-PerCPCy5.5 (53-6.7), CD11b-PE-Cy7 (M1/70), CD3-APC-Cy7 (17A2), Ly6C-PE (HK1.4), Ly6G-APC (1A8).
