# Supplementary material for: Clozapine reduces infiltration into the CNS by targeting migration in experimental autoimmune encephalomyelitis
Source: J Neuroinflammation. 2020 Feb 12;17:53. doi: 10.1186/s12974-020-01733-4 (PMC7014621; doi:10.1186/s12974-020-01733-4)
Supplement: Supplementary file 6 — Additional file 6:Figure S6. Gating strategy for CCR expression in blood. Gating strategy is shown for CCR expression analysis in blood (Fig. 5 and Additional file 4 a,b) from one EAE vehicle treated animal as an example, the following antibodies were used to detect the populations: CD4-BV521 (RM4–5), CD45-BV510 (30-F11), CD8-PerCPCy5.5 (53–6.7), CD11b-PE-Cy7 (M1/70), CD45R-AF488 (RA3-6B2), CCR2-PE (475301), CCR5-APC (HM-CCR5) Gr1-APC-Cy7 (RB6-8C5). [file 12974_2020_1733_MOESM6_ESM.pdf]

Supplement Figure 6

a

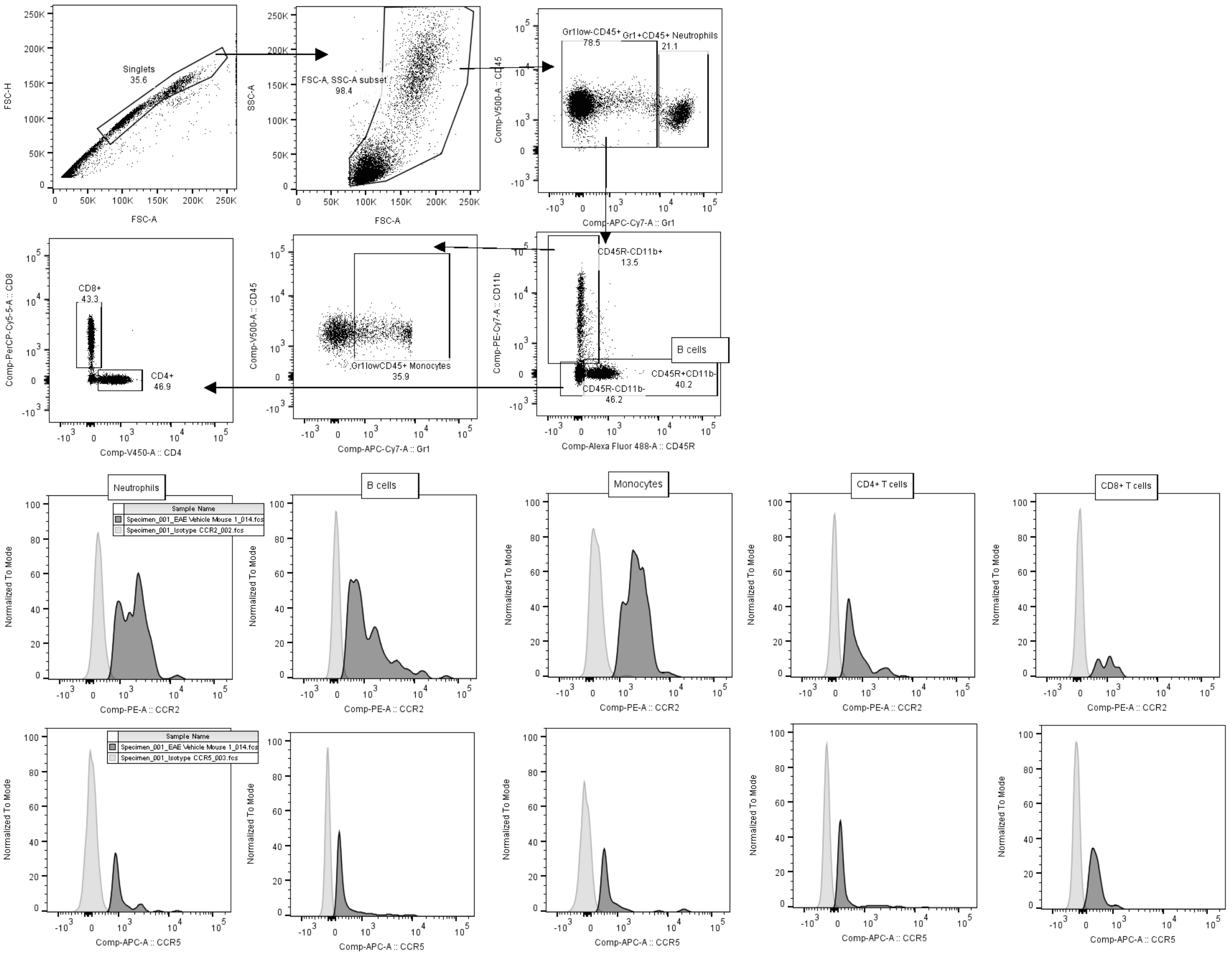

Additional file 6. Gating strategy for CCR expression in blood. Gating strategy is shown for CCR expression analysis in blood (Figure 5 and Additional file 4 a,b) from one EAE vehicle treated animal as an example, the following antibodies were used to detect the populations: CD4-BV521 (RM4-5), CD45-BV510 (30-F11), CD8-PerCPCy5.5 (53-6.7), CD11b-PE-Cy7 (M1/70), CD45R-AF488 (RA3-6B2), CCR2-PE (475301), CCR5-APC (HM-CCR5) Gr1-APC-Cy7 (RB6-8C5).
